# Treatment of Virulent Mycobacterium tuberculosis and HIV Coinfected Macrophages with Gallium Nanoparticles Inhibits Pathogen Growth and Modulates Macrophage Cytokine Production

**DOI:** 10.1128/mSphere.00443-19

**Published:** 2019-07-24

**Authors:** Seoung-ryoung Choi, Bradley E. Britigan, Prabagaran Narayanasamy

**Affiliations:** a Department of Pathology and Microbiology, College of Medicine, University of Nebraska Medical Center, Omaha, Nebraska, USA; b Department of Internal Medicine, College of Medicine, University of Nebraska Medical Center, Omaha, Nebraska, USA; c Research Service, VA Medical Center-Nebraska Western Iowa, Omaha, Nebraska, USA; Colorado State University

**Keywords:** *Mycobacterium tuberculosis*, gallium, human immunodeficiency virus, iron metabolism, nanoparticle

## Abstract

Tuberculosis (TB), caused by Mycobacterium tuberculosis, remains a global threat. The course of TB is negatively impacted by coexistent infection with human immunodeficiency virus type 1 (HIV). Macrophage infection with these pathogens modulates their production of pro- and anti-inflammatory cytokines, which could play a crucial role in pathogenesis. Despite the important role of macrophages in containing infection by a variety of microbes, both HIV and M. tuberculosis infect and replicate within these cells during the course of HIV-M. tuberculosis coinfection. Both M. tuberculosis and HIV require iron for growth and replication. We have previously shown that gallium encapsulated in nanoparticles, which interferes with cellular iron acquisition and utilization, inhibited the growth of HIV and an attenuated strain of M. tuberculosis within human monocyte-derived macrophages (MDMs) *in vitro*. Whether this was true for a fully virulent strain of M. tuberculosis and whether gallium treatment modulates cytokine production by HIV- and/or M. tuberculosis-infected macrophages have not been previously addressed. Therefore, coinfection of MDMs with HIV and a virulent M. tuberculosis strain (H37Rv) was studied in the presence of different gallium nanoparticles (GaNP). All GaNP were readily internalized by the MDMs, which provided sustained drug (gallium) release for 15 days. This led to significant growth inhibition of both HIV and M. tuberculosis within MDMs for up to 15 days after loading of the cells with all GaNP tested in our study. Cytokine analysis showed that HIV-M. tuberculosis coinfected macrophages secreted large amounts of interleukin 6 (IL-6) and IL-8 and smaller amounts of IL-1β, IL-4, and tumor necrosis factor alpha (TNF-α) cytokines. However, all GaNP were able to regulate the release of cytokines significantly. GaNP interrupts iron-mediated enzymatic reactions, leading to growth inhibition of HIV-M. tuberculosis coinfection in macrophages, and also modulates release of cytokines that may contribute to HIV-TB pathogenesis.

**IMPORTANCE** GaNP interrupts iron-mediated enzymatic reactions, leading to growth inhibition of virulent HIV-M. tuberculosis coinfection in macrophages, and also modulates release of cytokines that may contribute to HIV-TB pathogenesis. Macrophage-targeting GaNP are a promising therapeutic approach to provide sustained antimicrobial activity against HIV-M. tuberculosis coinfection.

## Abstract (precis)

GaNP interrupts iron-mediated enzymatic reactions, leading to growth inhibition of virulent HIV-M. tuberculosis coinfection in macrophages, and also modulates release of cytokines that may contribute to HIV-TB pathogenesis. Macrophage-targeting GaNP are a promising therapeutic approach to provide sustained antimicrobial activity against HIV-M. tuberculosis coinfection.

## INTRODUCTION

Mycobacterium tuberculosis, the causative agent of tuberculosis (TB), is one of the leading causes of mortality in the world. M. tuberculosis is more common in people living with human immunodeficiency virus type 1 (HIV). The World Health Organization (WHO) estimated that the risk of developing tuberculosis (TB) was 16 to 27 times higher in people living with HIV than among those without HIV infection in 2016 (1). Treatment of HIV-M. tuberculosis coinfection remains challenging despite tremendous efforts in the development of novel drugs against HIV and TB. HIV-M. tuberculosis coinfection requires prolonged multidrug treatment regimens, one to treat TB and the other to treat the underlying HIV infection. These regimens are complicated by drug toxicity, drug-drug interaction, and development of drug resistance (2). The emergence of multidrug-resistant (MDR) and extensively drug-resistant (XDR) TB necessitates identification of novel targets to develop new classes of antibiotics. To date, no single agent in clinical practice has been shown to inhibit both M. tuberculosis and HIV.

Macrophages, including alveolar macrophages, are reservoirs for both HIV and M. tuberculosis in humans. HIV-associated TB infections also accelerate the deterioration of the immune system via multiple mechanisms, leading to higher HIV replication, disruption of granuloma, and increased arrest of the development of M. tuberculosis phagosomes within macrophages (2, 3).

Cytokines and chemokines released by macrophages play a pivotal role in host defense via activation and recruitment of other immune cells to sites of microbial infection (4). M. tuberculosis-infected macrophages *in vitro* increase release of tumor necrosis factor alpha (TNF-α), interleukin-1β (IL-1β), IL-6, IL-8, and IL-12 (proinflammatory response). However, in the setting of HIV coinfection, M. tuberculosis-infected macrophages exhibit exaggerated increases in the release of these same proinflammatory cytokines (IL-6, IL-8, IL-1β, and TNF-α), as well as IL-10 and MIP-1α (5–7). HIV infection amplifies the risk for developing active TB by decreasing CD4^+^ and CD8^+^ T cells, resulting in rupture of granulomas comprised of M. tuberculosis-infected macrophages, dendritic cells, and T cells (8). HIV-mediated inhibition of IL-10 dysregulates innate immune responses to M. tuberculosis (9). These effects of HIV on the macrophage may in part be responsible for the poor response to treatment with TB drugs in patients with HIV-M. tuberculosis coinfection compared to TB patients not coinfected with HIV.

Iron (Fe) is an essential metal for most living organisms, including M. tuberculosis and HIV. Iron is involved in many enzymatic redox reactions because it readily cycles between ferrous (Fe^2+^) and ferric (Fe^3+^) oxidation states. In humans, there is little free iron available because iron is readily oxidized to produce insoluble ferric hydroxide under physiological conditions. In addition, iron is bound with high affinity by cellular proteins, such as hemoglobin, transferrin, lactoferrin, and ferritin, limiting iron accessibility to pathogenic microorganisms. Successful bacterial pathogens must have means to ensure adequate access to iron to grow and cause disease in an iron-limited host environment (10–12). For HIV, which usurps host cell enzymes (reverse transcriptase [RT]) for its replication, adequate iron within infected host cells is needed for optimal HIV replication. Hence, iron metabolism is a potential therapeutic target for new antimicrobial agents against both M. tuberculosis and HIV (13).

Gallium(III) (Ga) is a trivalent cationic element that shares similar properties with iron, including size, ionization potential, and electron affinity. Due to this similarity between the two metals, gallium is taken up by most prokaryotic and eukaryotic cells in biological systems similarly to iron. If simultaneously present with iron, gallium has the potential to disrupt iron uptake by competing with iron for acquisition by a cellular iron transport system(s). This results in cellular iron depletion. In addition, if cells take up gallium and insert it rather than iron into the active site of iron-centered enzymes (catalase and aconitase), the inability of Ga(III) to undergo reduction to Ga(II) results in a loss of enzymatic activity (14).

Several studies done by us and other research groups demonstrated that gallium disrupts iron acquisition by intracellular mycobacteria and Gram-positive and Gram-negative bacteria (11, 15, 16). Macrophage-targeted (mannose-tagged) nanoparticles have been developed to increase drug delivery to macrophages and demonstrated significant growth inhibition of mycobacteria relative to untreated controls (17–19). We also demonstrated that long-acting Ga nanoparticles (GaNP) inhibited the growth of both HIV and the attenuated H37Ra strain of M. tuberculosis during coinfection within human monocyte-derived macrophages (MDMs) *in vitro* (19–21). Treatment of the HIV-H37Ra coinfected macrophages by GaNP reduced the secretion of IL-6 and IL-8 for up to 15 days. However, whether these effects would also be seen with a fully virulent strain of M. tuberculosis, such as H37Rv, is not known. Here, we investigated the effect of GaNP on the treatment of HIV and virulent M. tuberculosis H37Rv coinfected macrophages and cytokine release from coinfected macrophages with/without GaNP. We have also synthesized rifampin-nevirapine nanoparticles from currently available drugs and compared their effect with that of GaNP.

## RESULTS

### Comparison of various MOIs of virulent M. tuberculosis residing in MDMs in HIV coinfection.

**FIG 1:**
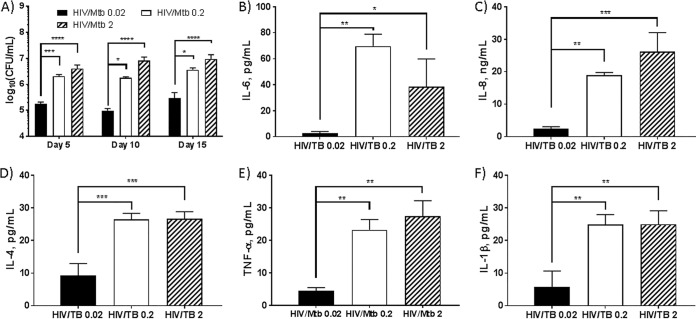
Comparison of virulent M. tuberculosis (MOIs of 0.02, 0.2, and 2) growth in MDMs coinfected with HIV-1 and cytokines released from coinfected MDMs. (A) MDMs were coinfected with HIV-1 (MOI = 0.01) and M. tuberculosis (H37Rv, MOI = 0.02, 0.2, or 2) for 24 h. After washing with PBS buffer to remove extracellular M. tuberculosis and HIV-1, the infected cells were incubated with change of medium every 24 h and lysed at days 5, 10, and 15 following infection to determine growth of M. tuberculosis in MDMs. (B to F) Cytokines released from coinfected MDMs. The supernatants were collected at day 5 and analyzed using the Luminex xMAP system. Data represent the mean ± standard deviation of the mean for triplicate wells. Statistically significant differences were determined using one-way ANOVA for multiple comparison (Tukey’s multiple comparisons, GraphPad Prism 7). *, *P* < 0.05; **, *P* < 0.01; ***, *P* < 0.001; ****, *P* < 0.0001.

MDMs were coinfected with HIV-1 (multiplicity of infection [MOI] = 0.01 viral particles per infection cell) and virulent M. tuberculosis strain H37Rv at 3 different multiplicities of infection (MOI = 0.02, 0.2, and 2) for 24 h and then incubated for up to 15 days, and CFU were determined after 2 to 3 weeks for the given day (5, 10, or 15). Increased growth of M. tuberculosis in MDMs was observed at all days when coinfected with higher numbers of M. tuberculosis (MOI = 0.2 and 2), and the growth acceleration may be due to higher M. tuberculosis MOIs. Also, there is no significant difference between M. tuberculosis infection at MOI = 0.2 and MOI = 2 at all days in the presence of HIV (Fig. 1A). Interestingly, when the M. tuberculosis MOI was 0.2, no significant change in M. tuberculosis growth was observed between M. tuberculosis infection alone and HIV-M. tuberculosis coinfection (data not shown), consistent with our previous results with nonpathogenic H37Ra (17, 18).

### The levels of cytokines released from coinfected MDMs were influenced by the degree of M. tuberculosis virulence.

We assessed cytokines secreted from MDMs coinfected with HIV and M. tuberculosis using a multiplex analysis. In our previous studies (17, 18), we demonstrated that MDMs coinfected with the attenuated strain M. tuberculosis H37Ra and HIV produced two major interleukins (IL-6 and IL-8), along with trace amounts of IL-1β, TNF-α, gamma interferon (IFN-γ), granulocyte-macrophage colony-stimulating factor (GM-CSF), and IL-4. Three interleukins (IL-2, IL-5, and IL-10) were not detected in H37Ra-infected macrophages, and none of the above 10 cytokines were observed in uninfected MDMs. However, MDMs coinfected with virulent H37Rv M. tuberculosis and HIV released large amounts of IL-6, IL-8, IL-1β, IL-4, and TNF-α (Fig. 1B to F). We found that the level of cytokines produced by HIV-M. tuberculosis coinfected MDMs increased with the magnitude of initial M. tuberculosis infection of the cells (MOI). Compared to an M. tuberculosis MOI of 0.02, MDMs inoculated with M. tuberculosis at the higher MOIs of 0.2 and 2 released IL-6 and IL-8 at 30-fold- and 10-fold-higher levels, respectively. Likewise, the higher M. tuberculosis MOIs stimulated the release of IL-4, IL-1β, and TNF-α by 3-, 5-, and 5-fold, respectively. Thus, we observed a different MDM cytokine profile when using the fully virulent H37Rv versus attenuated H37Ra in the setting of HIV coinfected MDMs. The results suggest that the cytokine profile produced by HIV-M. tuberculosis coinfected macrophages is affected by the relative virulence of the M. tuberculosis strain. In addition, when MDMs were coinfected with H37Rv at MOI = 0.2 and 2, no significant difference was observed in the given cytokines (IL-4, IL-6, IL-8, IL-1β, and TNF-α) released (Fig. 1B to F).

### Nanoparticle-treated MDMs inhibited HIV-M. tuberculosis coinfection.

In our previous study (17, 18), rifampin nanoparticles (RFPNP) and macrophage-targeted (mannose-tagged and folic acid-tagged) gallium nanoparticles were formulated and manufactured using a high-pressure homogenizer to investigate the effect of nanoparticles in inhibiting the growth of M. tuberculosis H37Ra residing in MDMs. We observed that GaNP provided sustained drug release for 15 days and exhibited significant growth inhibition against virulent M. tuberculosis H37Rv residing in macrophages (17).

**FIG 2:**
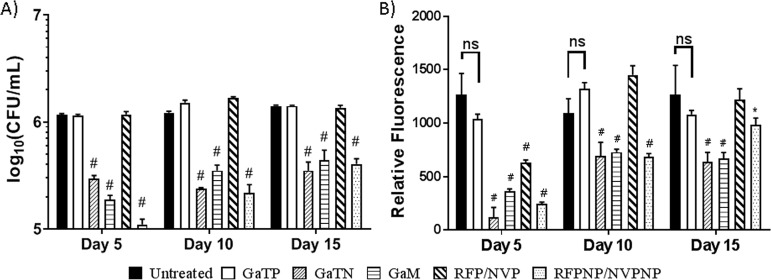
Prophylactic activities of gallium(III), rifampin, and nevirapine nanoparticles against virulent M. tuberculosis H37Rv and HIV-1 infected MDMs. (A) MDMs were pretreated with free drugs or their nanoparticles (300 μM) for 24 h followed by removal of extracellular drugs from the cells by washing. Pretreated MDMs were coinfected with M. tuberculosis (H37Rv, MOI = 0.2) and HIV-1 (MOI = 0.01) for 24 h at days 5, 10, and 15 following drug loading. After washing with PBS buffer to remove extracellular M. tuberculosis and HIV-1, the infected cells were incubated. After 2 days, the cells were lysed to determine level of M. tuberculosis in MDMs by CFU counting. (B) After 11 days, the supernatants were harvested to determine HIV growth inhibition by an RT assay. Data represent the mean ± standard deviation of the mean for triplicate wells. Statistically significant differences were determined using ANOVA (Tukey’s multiple comparisons, GraphPad Prism 7). *, *P* < 0.05; #, *P* < 0.0001, compared with the nontreated HIV-TB control. ns, no significance; GaTP, Ga tetraphenylporphyrin; GaTN, GaTP-encapsulated nanoparticle; GaM, mannose-tagged Ga nanoparticle encapsulating GaTP; RFP-NVP, combination of free drug rifampin and nevirapine; RFPNP-NVPNP, combination of rifampin nanoparticle and nevirapine nanoparticle.

Here, the effect of MDM loading with gallium(III) *meso*-tetraphenylporphyrin chloride (GaTP)-encapsulated nanoparticle (GaTN), mannose-tagged GaNP (GaM), nevirapine nanoparticles (NVPNP), or rifampin nanoparticles (RFPNP), as well as free drugs not incorporated into nanoparticles, on subsequent HIV-H37Rv coinfection was investigated. GaTN, GaM, and an RFPNP-NVPNP combination significantly reduced levels of both HIV and M. tuberculosis up to 15 days after MDMs were loaded with the nanoparticles (Fig. 2). Nevirapine and rifampin nanoparticles showed high drug uptake in MDM cells and no toxicity by MTT [3-(4,5-dimethyl-2-thiazolyl)-2,5-diphenyl-2H-tetrazolium bromide] and lactate dehydrogenase (LDH) assays ( see Text S1, Table S1, and Fig. S1 in the supplemental material). Interestingly, M. tuberculosis growth was reduced significantly compared to HIV growth on days 10 and 15. On day 15, GaTN and GaM showed significantly better HIV inhibition than the RFPNP-NVPNP combination. In contrast, free drugs (GaTP or RFP-NVP) failed to reduce the growth of HIV and M. tuberculosis, except for HIV growth reduction at day 5 for the RFP-NVP combination (Fig. 2B). In all cases, nanoparticles were more effective than free drugs in inhibiting M. tuberculosis and HIV replication.

10.1128/mSphere.00443-19.2 FIG S1 Cytotoxicity of nanoparticles. Cytotoxicity was determined using CytoTox96 nonradioactive cytotoxicity assay. GaTP, gallium tetraphenylporphyrin; NVPNP, nevirapine nanoparticle; RFPNP, rifampin nanoparticle; GaTN, GaTP-encapsulated nanoparticle. Download FIG S1, JPG file, 0.04 MB. Copyright © 2019 Choi et al. 2019 Choi et al. This content is distributed under the terms of the Creative Commons Attribution 4.0 International license.

10.1128/mSphere.00443-19.5 TABLE S1 Drug uptake by MDMs and viability. Download Table S1, DOCX file, 0.01 MB. Copyright © 2019 Choi et al. 2019 Choi et al. This content is distributed under the terms of the Creative Commons Attribution 4.0 International license.

10.1128/mSphere.00443-19.1 TEXT S1 Protocol for viability and cytotoxicity testing and quantitation of drug uptake by monocyte-derived macrophages (MDMs). Download Text S1, DOCX file, 0.02 MB. Copyright © 2019 Choi et al. 2019 Choi et al. This content is distributed under the terms of the Creative Commons Attribution 4.0 International license.

### Effect of HIV and M. tuberculosis coinfection in macrophages and the level of cytokines in culture supernatant: nanoparticles significantly reduce the levels of cytokines released from coinfected MDMs.

**FIG 3:**
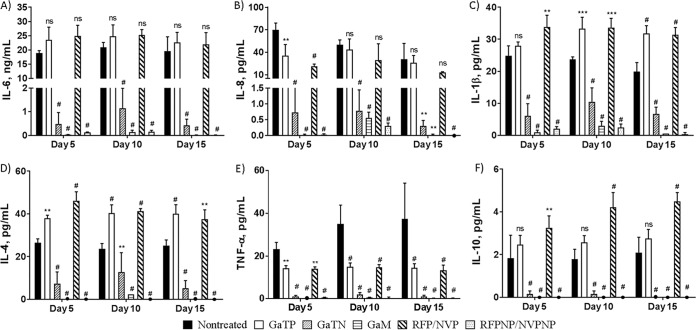
Analysis of cytokines produced by MDMs coinfected with HIV-1 and M. tuberculosis H37Rv. MDMs were coinfected with HIV-1 and M. tuberculosis (H37Rv) at days 5, 10, and 15 following drug treatment (300 μM). The culture supernatants were analyzed for the presence of cytokines released from infected MDMs on day 11 after infection. The data represent the mean ± standard deviation of the mean for 6 wells. Statistically significant differences were determined using one-way ANOVA (Tukey’s multiple comparisons, GraphPad Prism 7). **, *P* < 0.01; ***, *P* < 0.001; #, *P* < 0.0001, compared with the positive controls coinfected with HIV-1 and M. tuberculosis (HIV-M. tuberculosis). GaTP, free drug Ga tetraphenylporphyrin; GaTN, GaTP-encapsulated nanoparticle; GaM, mannose-tagged Ga nanoparticle encapsulating GaTP; RFP-NVP, combination of rifampin and nevirapine; RFPNP-NVPNP, combination of rifampin nanoparticle and nevirapine nanoparticle; ns, no significance.

In addition to determining the antimicrobial effect of these various drug-containing nanoparticles on HIV coinfection of macrophages (Fig. 2), we investigated their effect, as well as that of free drugs, on the release of cytokines from HIV-M. tuberculosis coinfected MDMs (Fig. 3). Ten cytokines were assessed from culture supernatants from MDMs coinfected with HIV and M. tuberculosis (H37Rv) by performing a multiplex analysis (Fig. 3). Only minor levels (<5 pg/ml) of the proinflammatory cytokines (IL-2, IFN-γ, and GM-CSF) and anti-inflammatory cytokines (IL-5) were detected (Fig. S3). All nanoparticle-treated MDMs produced significantly smaller amounts of the six cytokines (IL-6, IL-8, IL-1β, IL-4, IL-10, and TNF-α) compared to nontreated and free drug-treated MDMs, likely reflecting the anti-M. tuberculosis and anti-HIV activities of these nanoparticles.

Relative to untreated controls (infected with HIV and M. tuberculosis), GaTN and GaM decreased IL-6 and IL-8 by 40-fold and 70-fold, respectively, regardless of number of days after drug loading prior to infection. RFPNP-NVPNP also resulted in a significant drop (70-fold) in production of the same two cytokines. Interestingly here, GaM activity is found to be similar to RFPNP-NVPNP activity. In contrast, the production of cytokines measured was not affected by free drug GaTP or a combination therapy of free drug rifampin and nevirapine for many cytokines. In addition, GaTN and GaM decreased IL-1β, IL-4, IL-10, and TNF-α production significantly compared to the positive control. Only trace amounts of these four cytokines were detected from the supernatants of RFPNP-NVPNP- or GaM-treated macrophages. Thus, GaTN, GaM, or the combination of nevirapine and rifampin nanoparticles resulted in significant inhibition of the growth of HIV and M. tuberculosis and decreased many cytokine production levels even when infection of the MDMs occurred 15 days after drug loading, consistent with sustained drug release from the nanoparticles.

### GaTN exhibit antimicrobial and antiretroviral activities against M. tuberculosis and HIV preinfected macrophages.

**FIG 4:**
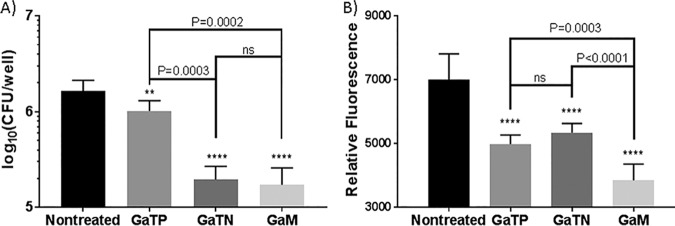
Antimicrobial and antiretroviral activities of Ga in MDMs coinfected with M. tuberculosis H37Rv and HIV-1. MDMs were coinfected with HIV-1 (MOI = 0.01 viral particles per infection cell) and M. tuberculosis H37Rv (MOI = 0.2) for 24 h. Coinfected MDMs were washed to remove extracellular M. tuberculosis and HIV and then treated with GaTP or Ga nanoparticles for 24 h (day 0). The cells were lysed at day 5 following treatment to determine growth of M. tuberculosis in MDMs by counting CFU (A). HIV growth inhibition was determined from supernatants from coinfected cells by a reverse transcriptase assay (B). Data represent the mean ± standard deviation of the mean for triplicate wells. Statistically significant differences were determined using one-way ANOVA (Tukey’s multiple comparisons, GraphPad Prism 7). **, *P* < 0.01, and ****, *P* < 0.0001, compared to untreated control; ns, no significance.

We next assessed the ability of the various nanoparticles and free drugs to alter the course of already established M. tuberculosis-HIV coinfection of MDMs. Thus, MDMs were first coinfected with HIV-H37Rv, which was followed the next day by drug treatment. Antimicrobial and antiretroviral activities of GaTN against HIV and M. tuberculosis coinfected MDMs were determined at day 5. As expected, GaM significantly inhibited growth of both HIV and M. tuberculosis within MDMs compared to free GaTP (Fig. 4). GaTN and GaM nanoformulations exhibited similar effectiveness against M. tuberculosis. Growth of intracellular M. tuberculosis decreased by 8-fold on day 5 with both GaTN and GaM compared to the untreated control. We also observed 20% and 25% reductions in replication of HIV after treatment with GaTN and GaM, respectively, compared to the untreated control. The results indicate that GaM inhibited the growth of both pathogens residing in preinfected macrophages.

## DISCUSSION

Iron is an essential nutrient for most living organisms. Iron is efficiently sequestered in the human host as part of a strategy of nutritional immunity (22, 23). Intracellular pathogens, like M. tuberculosis and HIV, encounter an iron-limited environment in the host. Intracellular pathogens have developed efficient iron uptake pathways to overcome and survive in iron-limited environments. Therefore, interrupting iron/heme uptake mechanisms in pathogens is a promising target for the development of antimicrobials.

We and others have previously shown that gallium complexes are able to interrupt iron/heme acquisition and growth of several different microorganisms. Ga(NO_3_)_3_, which is FDA approved for the treatment of hypercalcemia of malignancy (16, 24, 25), exhibits antimicrobial activity against *Francisella* sp., Pseudomonas aeruginosa, Acinetobacter baumannii, and mycobacteria within mononuclear phagocytes by disrupting Fe acquisition (11, 15, 26–28). Noniron metalloporphyrins, heme mimetics, also show growth inhibition against mycobacteria and Gram-positive and Gram-negative bacteria (29–31).

Our previous studies with GaTP and its nanoparticles demonstrated antimicrobial as well as antiretroviral activity against M. tuberculosis (H37Rv), M. tuberculosis (H37Ra), and HIV, as well as M. tuberculosis (H37Ra)-HIV and Mycobacterium smegmatis-HIV, residing in macrophages (17, 18). GaNP exhibited significant resistance to the growth of these intracellular pathogens for up to 15 days after the cells were loaded with the nanoparticles, suggesting sustained release of Ga(III) into the intracellular compartment. However, we had not previously determined whether the GaNP were also able to inhibit HIV-M. tuberculosis coinfection when a fully virulent form of M. tuberculosis, such as H37Rv, was used. Furthermore, we sought to explore the possibility of better targeting the GaNP with a ligand such as mannose that would be recognized by macrophage receptors, thereby enhancing uptake and efficacy.

Consistent with our previous results, as shown in Fig. 2 and 4, GaTN and GaM showed long-acting inhibitory activity against HIV and virulent M. tuberculosis H37Rv during simultaneous infection of MDMs with the two pathogens. The combined antimicrobial (rifampin and nevirapine) nanoparticles demonstrated sustained antimicrobial and antiretroviral drug activity against both pathogens (Fig. 2). Neither the combination of rifampin-nevirapine or GaTP alone when presented to MDMs in its “free drug” form demonstrated growth inhibition of HIV-M. tuberculosis either at day 10 postincubation or beyond. It is likely that these free drugs did not remain inside the macrophage at levels needed to inhibit HIV or M. tuberculosis after 6 days. In contrast, the nanoformulations of both gallium and rifampin-nevirapine exhibited excellent inhibition of both M. tuberculosis and HIV even up to 15 days after loading of the MDM, confirming the therapeutic advantages of nanoformulation compared to traditional free drug.

HIV infection dysregulates cytokine production of T cells and macrophages, the main targets of HIV, resulting in defective immunological function (32, 33). HIV infection reportedly increases IL-6, IL-1, IL-8, IL-4, IL-10, and TNF-α productions and decreases Th1 cytokines (IL-2 and IFN-γ) in MDM cells (34). M. tuberculosis infection in MDMs induces increased production of IFN-γ, TNF-α, IL-12, IL-8, and IL-6 (35–39). IFN-γ produced by T cells activates macrophages, which in turn produce reactive oxygen and nitric oxide species and release other cytokines and chemokines (3).

We evaluated the *in vitro* effect of GaTN on cytokines released by MDMs infected with HIV and M. tuberculosis H37Rv. As shown in Fig. 1 and 3, HIV-M. tuberculosis coinfected macrophages released two major (IL-6 and IL-8) and three minor (IL-1β, IL-4, and TNF-α) cytokines. However, only IL-6 and IL-8 were released in significant amounts from HIV-H37Ra coinfected MDMs (18).

**FIG 5:**
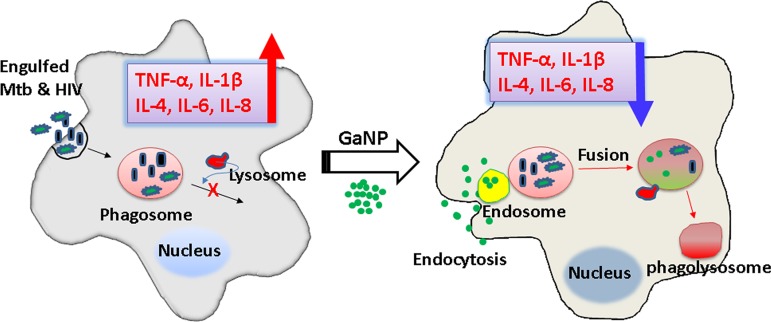
M. tuberculosis-HIV coinfection increases the amount of cytokines released from MDMs in comparison to uninfected MDMs, and GaNP treatment decreases the amount of cytokine release in comparison to infected MDMs.

We also observed that MDMs coinfected with HIV-M. tuberculosis H37Rv produced increased amounts of TNF-α, IL-1β, and IL-4 in response to M. tuberculosis infection at high MOIs (Fig. 1 and 5). M. tuberculosis-infected macrophages also increased TNF-α production, which is reported to contribute to macrophage activation, apoptosis, and granuloma formation that in turn play a role in limiting M. tuberculosis and HIV infection. Work by others has shown that M. tuberculosis inhibits macrophage apoptosis, which plays an important role in host defense against intracellular pathogens, by reducing production or negating the protective effects of TNF-α on macrophages in response to M. tuberculosis infection (40–43). Likewise, minimal TNF-α (<5 pg/ml) was detected when MDMs were infected with a low MOI (0.02) of M. tuberculosis. However, 4- to 5-fold-higher TNF-α levels were detected with the highest MOI for virulent M. tuberculosis (Fig. 1E).

In contrast, in a circumstance of coinfection with HIV and M. tuberculosis at a high MOI, the intracellular pathogens may induce macrophage necrosis by allowing TNF-α production from infected macrophages, by which the pathogens could spread to uninfected macrophages by phagocytosis (44).

Proinflammatory IL-1 (IL-1α and IL-β), IL-6, and IL-8 are cytokines released from macrophages in response to infection, including that with HIV and M. tuberculosis. These cytokines also play fundamental roles in host defense. Consistent with our prior study with coinfection of MDMs with HIV and the attenuated H37Ra strain of M. tuberculosis, significantly larger amounts of IL-6 and IL-8 were found in supernatants from MDMs coinfected with HIV and H37Rv compared to uninfected MDMs (Fig. 1). Similarly to TNF-α, a smaller amount of IL-1β was detected compared to IL-6 and IL-8. Recent reports suggest that IL-6 may inhibit production of TNF-α and IL-1β and macrophage responses to IFN-γ and thereby promote M. tuberculosis growth, which was also observed in our study (45–47). HIV infection also increases proinflammatory cytokines (IL-1, IL-6, and IL-8) (48) and anti-inflammatory cytokines IL-4 and IL-10 (49), which may contribute to detection of slightly increased levels of IL-1β and IL-4 from MDMs coinfected with HIV-H37Rv. Similarly, we also observed slightly increased levels of IL-1β and IL-4 from MDMs coinfected with HIV-H37Rv.

As shown in Fig. 3, the production of all five cytokines were significantly reduced in culture supernatants from GaTN-treated MDMs coinfected with HIV and H37Rv compared to untreated MDMs (Fig. 5). As described before (17), this reduction may be due to enhanced phagolysosome formation because of GaTN. Similarly, it could involve modulation of the IκB kinase-β/NF-κB pathway in HIV-H37Rv coinfection, as reported earlier (18). The RFP-NVP nanoparticles also reduced the cytokines from HIV-H37Rv coinfected MDMs. However, free-drug-treated MDMs failed to inhibit growth of both pathogens, suggesting that no significant amount of free drugs remained in MDMs after day 6 posttreatment.

### Conclusion.

Ga porphyrins and Ga(III) nanoparticles interrupt iron/heme acquisition by pathogens. GaTN and GaM showed prolonged antimicrobial and antiretroviral activity following loading of MDMs prior to HIV-M. tuberculosis infection, as well as the ability to inhibit established HIV-M. tuberculosis infection. Their efficacy was similar to that seen with nanoparticles of rifampin and nevirapine. Coinfection of human MDMs with HIV and H37Rv resulted in a different cytokine profile than we previously reported with HIV-M. tuberculosis coinfection using the attenuated H37Ra strain of M. tuberculosis. This suggests that M. tuberculosis virulence factors may significantly modulate the inflammatory response of M. tuberculosis-infected macrophages. HIV-1-H37Rv coinfected MDMs *in vitro* exhibited increased production of IL-6, IL-8, IL-1β, IL-4, and TNF-α. GaTN-treated MDMs showed reduced levels of these 5 cytokines relative to nontreated control HIV-M. tuberculosis coinfected macrophages. GaTN and GaM reduced both M. tuberculosis growth and replication of HIV in precoinfection and postcoinfection MDMs. These studies support further development of GaNP as novel antimicrobial agents that provide the ability to inhibit M. tuberculosis and HIV infection of macrophages with a single drug.

## MATERIALS AND METHODS

### Reagents.

Gallium(III) *meso*-tetraphenylporphyrin chloride (GaTP) was purchased from Frontier Scientific (Logan, UT). THP-1 cells were purchased from ATCC. Difco Middlebrook 7H9 broth and BBL Middlebrook oleic acid-albumin-dextrose-catalase (OADC) enrichment medium were purchased from BD (Sparks, MD, USA). 7H10 agar plates were purchased from Remel Inc. (Lenexa, KS). HyClone RPMI 1640, Dulbecco modified Eagle medium (DMEM), l-glutamine, HEPES, sodium pyruvate, and fetal bovine serum were purchased from GE Life Sciences (Logan, UT). Macrophage colony-stimulating factor (MCSF) was purchased from BioLegend (San Diego, CA). Fe-free 7H9 medium was prepared as described previously (11). Human monocytes that had been purified by countercurrent centrifugal elutriation from normal human donors were purchased from the UNMC Elutriation core facility using an institutional IRB-approved protocol. HIV-1_ADA_ was obtained from UNMC, Omaha, and its potency was tested by reverse transcriptase (RT) assay before infection. H37Rv was obtained from ATCC and grown in a biosafety level 3 (BSL3) lab, and its potency was tested by counting CFU before infection.

### Preparation and characterization of GaTN and rifampin nanoparticles.

GaTP-encapsulated nanoparticles (GaTN) and rifampin nanoparticles were formulated from gallium(III) *meso*-tetraphenylporphyrin or rifampin, manufactured using a high-pressure homogenizer (Avestin Inc., Ottawa, ON, Canada), and characterized by dynamic light scattering (DLS) and scanning electron microscopy (SEM) as previously described (17–20, 50). In brief, the mixture of 1% (wt/vol) GaTP or rifampin, 0.5% F127 polymer, and 0.5% sucrose in 20 ml of 10 mM HEPES solution was stirred overnight at room temperature. The mixture was homogenized at 20,000 lb/in^2^ until consistent size and polydispersity were attained. The nanoparticles were washed three times with 10 mM HEPES by centrifugation at 10,000 rpm at 4°C for 30 min. The pellets were resuspended in 10 mM HEPES and stored at 4°C (17).

### Preparation of NVPNP.

Nevirapine nanoparticles (NVPNP) were synthesized using F127 polymer and NVP by high-pressure homogenization as described above (see Fig. S2 in the supplemental material) (17). NVPNP were characterized using DLS and SEM. The size of the nanoparticles was 190 ± 0.7 nm with a polydispersity index (PDI) of 0.3 ± 0.052, and the zeta potential was −7.3 ± 0.1. The SEM image indicated that NVPNP have a rough rectangular shape and are smaller than the Ga(III) and rifampin nanoparticles, as reported in our previous studies (17).

10.1128/mSphere.00443-19.3 FIG S2 SEM image of nevirapine nanoparticles. High-pressure homogenization was used to formulate the nanoparticles from F127 and nevirapine in 1 mM HEPES buffer. Download FIG S2, JPG file, 0.01 MB. Copyright © 2019 Choi et al. 2019 Choi et al. This content is distributed under the terms of the Creative Commons Attribution 4.0 International license.

10.1128/mSphere.00443-19.4 FIG S3 Analysis of cytokines produced by MDMs coinfected with HIV-1 and M. tuberculosis H37Rv. MDMs were coinfected with HIV-1 and M. tuberculosis (H37Rv) at days 5, 10, and 15 following drug treatment (300 μM). The media were replaced on day 9 after infection. Supernatants were analyzed for the presence of cytokines released from infected MDMs on day 11 after infection. The data represent the mean ± standard error of the mean for 6 wells. Statistically significant differences were determined using ANOVA (GraphPad). ****, *P* < 0.0001, compared with the positive controls coinfected with HIV-1 and M. tuberculosis (HT). GaTP, free drug Ga tetraphenylporphyrin; GaNP, Ga nanoparticle; GaM, mannose-tagged Ga nanoparticle; RFP-NVP, free drug rifampin and nevirapine; RFPNP-NVPNP, rifampin nanoparticle and nevirapine nanoparticle. Download FIG S3, JPG file, 0.06 MB. Copyright © 2019 Choi et al. 2019 Choi et al. This content is distributed under the terms of the Creative Commons Attribution 4.0 International license.

### Coinfection of human MDMs with M. tuberculosis (H37Rv) and HIV-1 without Ga treatment.

Human monocytes (0.75 × 10^6^ cells/well/ml) were differentiated into MDMs in DMEM supplemented with 10% heat-inactivated pooled human serum (Innovative Biologics, Herndon, VA, USA), 50 μg/ml gentamicin (Mediatech Inc., Manassas, VA), 10 ng/ml MCSF, and 10 mM sodium pyruvate at 37°C in a 5% CO_2_ humidified atmosphere. On the 5th day of incubation and then every 2 days thereafter, half the medium was replaced. At the 10th day, the medium was replaced with DMEM supplemented with 1% human serum and the differentiated MDMs were incubated for 24 h. On day 11, for HIV-M. tuberculosis coinfection studies, the MDMs were incubated with HIV-1 (MOI = 0.01, viral particles per infection cell) and H37Rv (MOI = 0.02, 0.2, or 2) for 24 h in DMEM supplemented with 1% human serum without gentamicin. After infection, the MDMs were washed with phosphate-buffered saline (PBS) to remove extracellular M. tuberculosis and HIV, and the cells were incubated in DMEM supplemented with 1% human serum for 5, 10, or 15 days with medium replacement every 2 day. The supernatants were collected to analyze cytokines, and the infected macrophages were washed with PBS three times and lysed for the determination of M. tuberculosis CFU within MDMs. Iced sterile water (300 μl) was added to the wells followed by incubation on ice for 10 min. To the wells was added 1.2 ml of lysis buffer containing 55% 7H9 broth, 20% of 0.25% SDS, and 25% of 20% bovine serum albumin (BSA) in PBS. The lysed cells were centrifuged at 14,000 × *g* for 15 min. The pellets were resuspended in 200 μl of PBS, serially diluted in sterile PBS, and plated onto 7H10 agar plates for determination of M. tuberculosis CFU after 2 to 3 weeks.

### Quantitation of cytokines.

The human cytokines (GM-CSF, IFN-γ, IL-1β, IL-2, IL-4, IL-5, IL-6, IL-8, IL-10, and TNF-α) present in culture supernatants were analyzed using a multiplex kit (Invitrogen human cytokine 10-plex panel; Camarillo, CA) according to the manufacturer’s instructions. Fluorescence was measured using the Luminex xMAP system (Athena Multi-Lyte; Bio-Rad, USA). All samples were assayed in triplicate, with standards run in duplicate.

### Determination of M. tuberculosis and HIV growth in pretreated MDMs.

At the 11th day of monocyte differentiation as described above, MDMs (0.75 × 10^6^ cells/well/ml, 24-well plate) were treated with free drugs or their nanoparticles (300 μM for monotherapy, 150 μM each for combination therapy) suspended in DMEM supplemented with 1% human serum for 24 h at 37°C in a 5% CO_2_ humidified atmosphere for postinfection studies. After 24 h of treatment, drug-treated MDMs or control MDMs were washed with PBS three times and incubated in DMEM (1% human serum) before infection. The drug-treated MDMs were infected with both M. tuberculosis H37Rv (MOI = 0.2) and HIV-1 (MOI = 0.01) at days 5, 10, and 15 following drug loading and washed, fresh medium was added, and cells continued in culture for 2 days. The MDMs were washed with PBS three times and lysed for the determination of M. tuberculosis CFU residing in MDMs as described above.

For the determination of HIV growth and cytokine analysis in pretreated MDMs, drug-treated MDMs (0.75 × 10^6^ cells/well/ml, 24-well plate) were infected with both HIV-1 (MOI = 0.01) and M. tuberculosis H37Rv (MOI = 0.2) at days 5, 10, and 15 following drug loading for 24 h and washed, new medium was added, and cells continued in culture for 11 days at 37°C, with replacement of the DMEM (1% human serum) every 2 days. The culture supernatants were collected at day 11 postinfection to determine HIV growth and analyze cytokines released from coinfected MDMs. A reverse transcriptase (RT) activity assay (EnzChek; Molecular Probes, Eugene, OR) was performed, as described in the manufacturer’s protocol, to determine the magnitude of HIV present in the MDM cultures. The human cytokines (GM-CSF, IFN-γ, IL-1β, IL-2, IL-4, IL-5, IL-6, IL-8, IL-10, and TNF-α) present in culture supernatants were analyzed as described above.

### Antimicrobial and antiretroviral activities of GaNP in MDMs preinfected with M. tuberculosis (H37Rv) and HIV-1.

MDMs (0.75 × 10^6^ cells/well/ml, 24-well plate) were initially coinfected with HIV-1 (MOI = 0.01) and H37Rv (MOI = 0.2) for 24 h in DMEM supplemented with 1% heat-inactivated human serum (no antibiotics). After washing with PBS buffer, the cells were treated with GaTP or GaNP (300 μM) for 24 h. Extracellular Ga was removed by washing the cells with PBS three times. The treated MDMs were incubated in DMEM supplemented with 1% heat-inactivated human serum. At day 5 (the preinfected cells on the 10th day were not healthy) following infection, the treated MDMs were lysed to determine M. tuberculosis CFU, as described above. The culture supernatants were used to quantitate the HIV present by RT activity assay, as described above.

### Statistical analysis.

Statistical analysis was performed using one-way analysis of variance (ANOVA) for multiple comparison (GraphPad Prism 7). Data are presented as mean ± SD. Data are considered significant at *P* < 0.05.
