# Supplementary figures and images for: Treatment of Virulent Mycobacterium tuberculosis and HIV Coinfected Macrophages with Gallium Nanoparticles Inhibits Pathogen Growth and Modulates Macrophage Cytokine Production
Source: mSphere. 2019 Jul 24;4(4):e00443-19. doi: 10.1128/mSphere.00443-19 (PMC6656872; doi:10.1128/mSphere.00443-19)

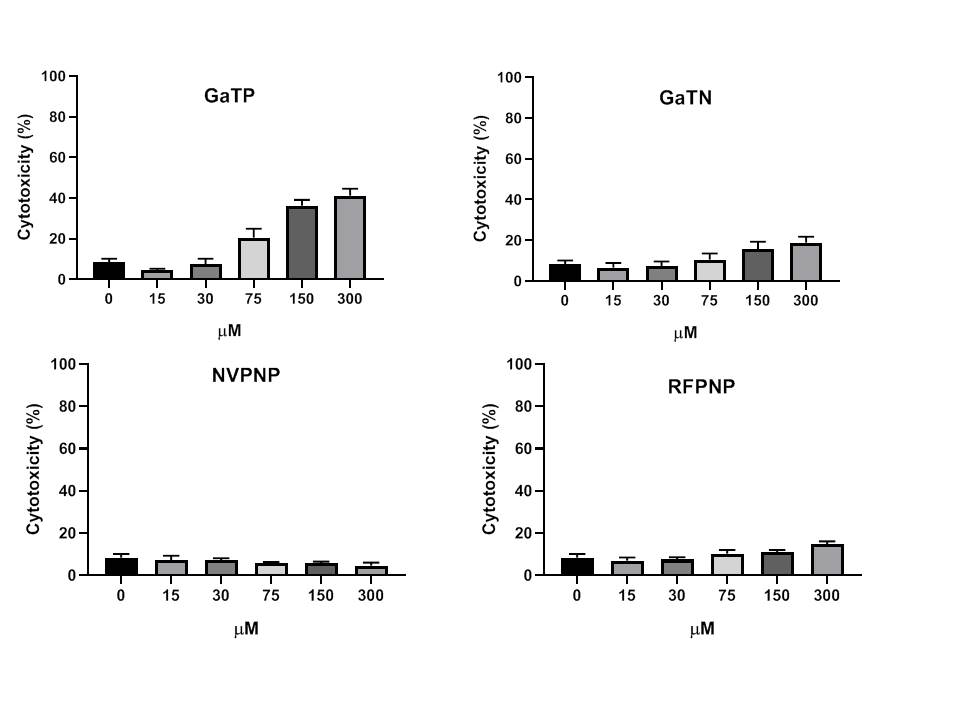

Supplement: FIG S1 [file mSphere.00443-19-sf001.jpg]

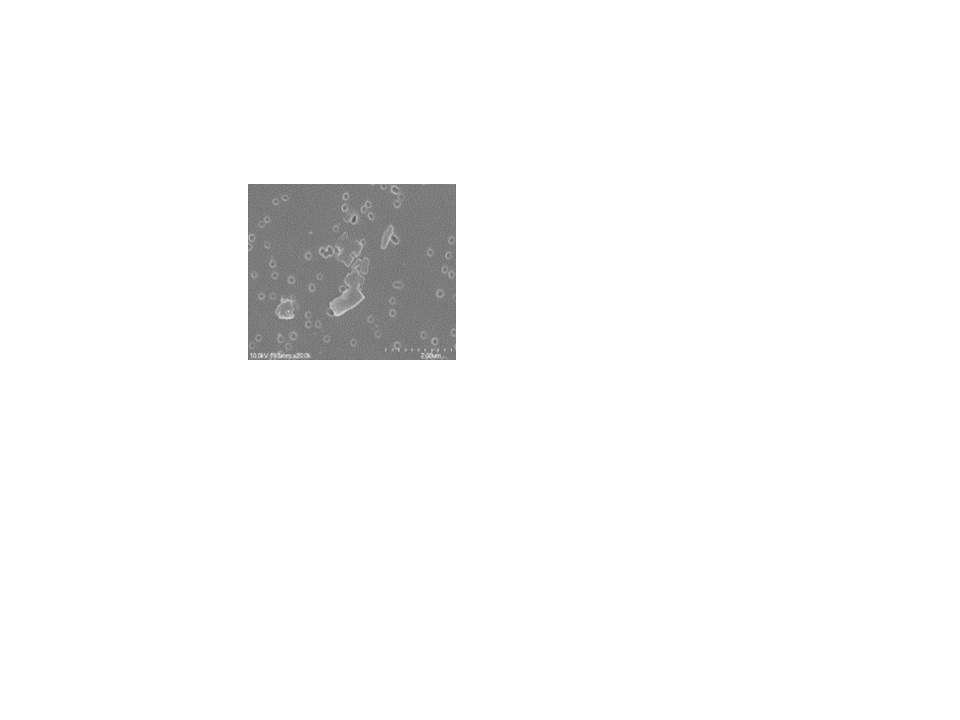

Supplement: FIG S2 [file mSphere.00443-19-sf002.jpg]

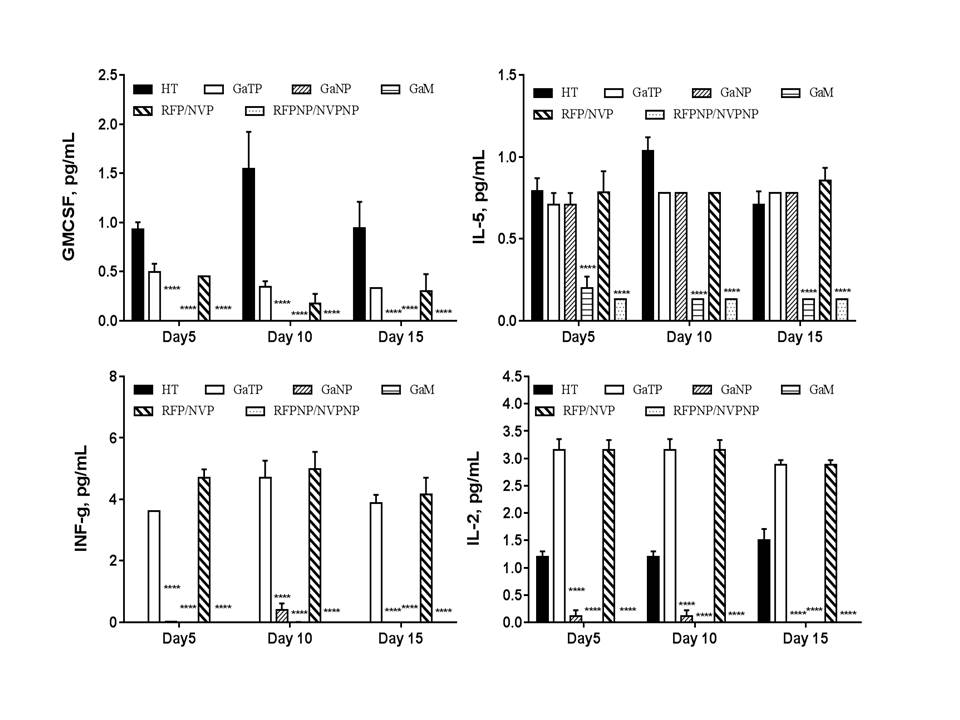

Supplement: FIG S3 [file mSphere.00443-19-sf003.jpg]
