# Supplementary material for: Treatment of Virulent Mycobacterium tuberculosis and HIV Coinfected Macrophages with Gallium Nanoparticles Inhibits Pathogen Growth and Modulates Macrophage Cytokine Production
Source: mSphere. 2019 Jul 24;4(4):e00443-19. doi: 10.1128/mSphere.00443-19 (PMC6656872; doi:10.1128/mSphere.00443-19)
Supplement: TABLE S1 [file mSphere.00443-19-st001.docx]

|  | Drug uptake | Cell viability (MTT assay) |
| --- | --- | --- |
| Nevirapine nanoparticle (300 µM) | 1.6 µg/10^6^ cells at 5 h of incubation | 100 % |
| Rifampin nanoparticle (300 µM) | 20 µg/10^6^ cells at 20 h of incubation | 100 % |
