# Supplementary material for: Treatment of Virulent Mycobacterium tuberculosis and HIV Coinfected Macrophages with Gallium Nanoparticles Inhibits Pathogen Growth and Modulates Macrophage Cytokine Production
Source: mSphere. 2019 Jul 24;4(4):e00443-19. doi: 10.1128/mSphere.00443-19 (PMC6656872; doi:10.1128/mSphere.00443-19)
Supplement: TEXT S1 [file mSphere.00443-19-s0001.docx]

**Treatment of Virulent *Mycobacterium tuberculosis* and HIV Coinfected Macrophages with Gallium Nanoparticles Inhibits Pathogen Growth and Modulates Macrophage Cytokine Production**

*Seoung-ryoung Choi^1^, Bradley E. Britigan^1,2,3^ and Prabagaran Narayanasamy^1,*^*

**^1^**Department of Pathology and Microbiology, **^2^**Department of Internal Medicine, College of Medicine, University of Nebraska Medical Center, Omaha, Nebraska 68198 and **^3^**Research Service, VA Medical Center-Nebraska Western Iowa, Omaha, Nebraska 68105.

PN – p.narayanasamy@unmc.edu

**Text S1.**

# **Viability and Cytotoxicity.** Cell viability was determined by MTT assay. In brief, monocyte-derived macrophages (MDM) were treated with individual compound or nanoparticles at a concentration of 300 µM for 24 h. The treated cells were then washed with PBS three times to remove extracellular compounds. MTT (5 mg/mL) was added and the cells were incubated for 30 min at 37°C. After washing with PBS, DMSO was added and the cells were incubated for 15 min at room temperature. Absorbance at 490 nm was quantitated using Biotek Synergy H1 hybrid Reader. Cell cytotoxicity was measured using CytoTox96^®^ Non-Radioactive assay from Promega (Madison, WI) according to the manufacturer’s protocol. In brief, THP-1 macrophages were treated with nanoparticles at various concentrations for 24 h. Absorbance signal was measured at 490 nm using Biotek Synergy H1 hybrid Reader.

**Quantitation of drug uptake by monocyte-derived macrophages (MDM)**. MDM uptake of nanoparticles was determined as described previously.^1^ In brief, MDMs were treated with 300 µM nanoparticles for 24 h. After washing extracellular nanoparticles with PBS buffer (x3), MDM uptake of nanoparticles was determined at 5 h and 20 h of incubation. The MDMs were scraped into 1 mL PBS and pelleted by centrifugation at 500 x g for 10 min at 4 °C. The pellets were resuspended in 200 µL methanol, sonicated, and centrifuged at 20000 x g for 10 min at 4 °C. The methanol extract was analyzed to determine drug quantity using HPLC as described.^1^

**Scanning electron microscopy (SEM)**. SEM images of nevirapine nanoparticles (Figure S2) was obtained using a Hitachi S4700 Field-Emission Scanning Electron Microscope (Hitachi High Technologies America, Inc., Schaumburg, IL, USA).^2^

**Reference**.

1. Edagwa, B. J.; Guo, D.; Puligujja, P.; Chen, H.; McMillan, J.; Liu, X.; Gendelman, H. E.; Narayanasamy, P., Long-acting antituberculous therapeutic nanoparticles target macrophage endosomes. *FASEB J* **2014**.

2. Balkundi, S.; Nowacek, A. S.; Veerubhotla, R. S.; Chen, H.; Martinez-Skinner, A.; Roy, U.; Mosley, R. L.; Kanmogne, G.; Liu, X.; Kabanov, A. V.; Bronich, T.; McMillan, J.; Gendelman, H. E., Comparative manufacture and cell-based delivery of antiretroviral nanoformulations. *Int J Nanomedicine* **2011,** *6*, 3393-404.
